# Effect of Femoral Head and Neck Osteotomy on Canines’ Functional Pelvic Position and Locomotion

**DOI:** 10.3390/ani12131631

**Published:** 2022-06-24

**Authors:** Madeleine Engstig, Senni Vesterinen, Mikael Morelius, Jouni Junnila, Heli K. Hyytiäinen

**Affiliations:** 1 School of Veterinary Science, University of Liverpool, Leahurst Campus, Chester High Road, Neston CH64 7TE, Wirral, UK; madeleine.engstig@evidensia.se; 2 Department of Equine and Small Animal Medicine, Faculty of Veterinary Medicine, University of Helsinki, Viikintie 49, 00014 Helsinki, Finland; senni.s.vesterinen@gmail.com (S.V.); mikael.morelius@sipoonpienelainklinikka.fi (M.M.); 3 EstiMates Oy, Tykistökatu 4, 20520 Turku, Finland; jouni.junnila@estimates.fi

**Keywords:** physiotherapy, rehabilitation, function, locomotion, coxofemoral joint

## Abstract

**Simple Summary:**

Removal of the femoral head and neck is a common surgical procedure used to provide pain relief in dogs with severe hip-related diseases. We investigated the chronic effect of the surgery on movement and position of the pelvis. Dog owners completed a questionnaire regarding their dog’s rehabilitation. An orthopaedic examination, anatomical measurements, and pressure-sensitive walkway analysis were conducted on all of the dogs. According to the questionnaire results, nine of ten dogs had returned to normal physical activity level, and the owners of the dogs considered the outcome of the surgery to be good or excellent. However, the dogs had less muscle mass, less hip extension, and less weight bearing on the operated limb when standing. The trot of the dogs was unaffected; no differences emerged between the operated and non-operated limb regarding length of stance or swing time of step or weight bearing during step. Pelvic position did not change in a specific way; i.e., the pelvis did not tilt towards or away from the surgically treated limb.

**Abstract:**

The long-term effect of femoral head and neck osteotomy (FHO) on the locomotory system of dogs was evaluated. The study comprised an owner questionnaire and an orthopaedic examination, anatomical measurements, and pressure-sensitive walkway analysis for dogs. Linear mixed effect models were used for statistical analysis. Ten dogs with a median of 2.5 years since their unilateral FHO were included. According to the questionnaire results, nine dogs had returned to a normal physical activity level. Muscle atrophy (*p* = 0.005), less extension in the coxofemoral joint (*p* = 0.003), and less static weight bearing on the FHO limb (*p* = 0.003) were observed. No consistent pattern regarding tilt or position of the pelvis was noted when measuring height of the tuber ischii (*p* = 0.39). Five of the dogs tilted away from, and five towards the FHO side when measured from the tuber sacrale with a Myrin goniometer. No differences regarding stance time, swing time, or peak pressure between the FHO and non-FHO limb were seen in trot (*p* = 0.70, *p* = 0.26, and *p* = 0.91, respectively). Over the long term, the FHO limb has muscle atrophy, decreased coxofemoral extension, and decreased static weight bearing. However, this does not seem to affect the trot of the dogs. Dog owners considered the outcome of surgery to be good or excellent.

## 1. Introduction

There are several approaches to surgically treat coxofemoral diseases in dogs and cats [1]. Total hip replacement has become an accepted veterinary orthopaedic procedure for a variety of disorders and often considered gold standard [2,3]. Other contemporary options include juvenile pubic symphysiodesis, double pelvic osteotomy or triple pelvic osteotomy in younger dogs with hip dysplasia [4,5]. The above-mentioned approaches are considered as primary surgical treatment options. However, in cases with, for example limited financial recourses, femoral head and neck osteotomy (FHO) can be considered as an acceptable second option surgical procedure [6]. It is used to provide pain relief in patients with coxofemoral joint-related diseases, such as severe hip dysplasia and advanced osteoarthritis, or for indications such as comminute or complicated fractures of the femoral head, neck or acetabulum where primary repair is not feasible, avascular necrosis of the femoral head such as Legg-Calvé-Perthes disease or chronic or recurrent coxofemoral luxations [1,7].

During FHO, the femoral head and neck is removed by excision of the neck from the base of the trochanter major across the neck in a line that intersects the medial cortex of the femur. Care should be taken not to include trochanter minor in the osteotomy line and aim for a smooth surface without irregularities or bone spurs [1]. Aims of the surgery are to limit contact between the femoral head and the acetabulum and to allow formation of dense fibrous tissue leading to a false joint or pseudoarthrosis [6]. As the surgery is non-reversible [1], it is often described as a salvage procedure to relieve pain and improve the quality of life once other conservative and/or surgical therapies have failed. However, it is often the first choice of treatment, especially after complicated fractures or necrosis of the femoral head [1]. Early return to function is crucial to adapt the fibrous tissue that forms during the post-operative period and build up a functional pseudoarthrosis with a good range of motion [6]. Appropriate post-operative physical therapy can improve the recovery [8] and provide a better outcome [8,9]. The early phase of post-operative rehabilitation aims to relieve pain and swelling, control inflammation, and promote tissue regeneration. The next phase aims to improve proprioception, weight bearing and maintain or improve the range of motion of the hip. The focus of the last phase of rehabilitation is strengthening of the hind quarter’s musculature [10,11].

The prognosis for a functional recovery after FHO is considered good [11]. According to previously published owner-reported data, the outcome of FHO is excellent [12]. Several studies have reported good or excellent owner satisfaction rates in 93–96% of cases [13,14,15]. There is, however, a discrepancy when comparing questionnaire results with objective functional measures [15]. Most of the dogs who have undergone FHO surgery do not regain completely normal gait [12]. Signs that have been reported to remain years after the FHO are limb shortening due to caudodorsal or craniodorsal malplacement of the proximal femur during weight bearing in 62–83% of dogs [12,13,15], muscle atrophy in 50–82% of dogs, and reduced range of motion in 74% and lameness in 30–68% of dogs [13,15]. Tilted pelvic position away from the FHO limb has been reported in 38% of dogs [13]. A shortened stance time on the FHO limb has been described in both small and large dogs [15], as well as decreased peak propulsive force and vertical impulse [16], and continued pain and reduced exercise tolerance [7].

FHO was first described and referred to in 1956 [13] and remains a common procedure despite of the clear discrepancy between owner-reported outcome and objective measurements. Studies regarding post-operative physiotherapy programmes following FHO are scarce [9], although physiotherapy is often suggested [7,8,17,18], and points in a positive direction with improved outcome in the few studies conducted [9,19]. It is therefore important to continue to investigate the effect of the surgery and how we can further improve the outcome with post-operative physiotherapy.

The aim of this study was to examine clinical signs, pelvic position, and locomotion in dogs who had undergone FHO at least 6 months earlier and received post-operative physiotherapy. We hypothesized that there was less thigh circumference, decreased coxofemoral extension, and decreased weight bearing in the FHO limb compared with the contralateral limb. Our second hypothesis was that the pelvis would have been dorsally tilted away from the FHO side, i.e., higher on the FHO side. Further, we suggested that the stance time, swing time, peak pressure, and step length of the FHO limb would have been decreased relative to the contralateral limb. 

## 2. Materials and Methods

This study was conducted under the permission of the National Experiment Board in Finland (ESAVI/5794/04.10.03/2011), Helsinki, Finland. 

Ten dogs that had undergone FHO at the University of Helsinki Veterinary Teaching Hospital and attended physiotherapy post-operatively participated in the study. Post-operative physiotherapy was routinely recommended following FHO surgery. Data collection was conducted prospectively in October 2013 and March 2014. The inclusion criterion was unilateral FHO, performed at least six months before the study. Exclusion criteria were bilateral FHO, coxofemoral replacement in the contralateral limb, or any other orthopaedic or neurological findings in back, front, and hind limb structures not directly related to the coxofemoral joint that was considered painful or affecting the gait. The study consisted of an owner questionnaire and orthopaedic examination, anatomical measurements, and pressure-sensitive walkway analysis, performed in that same order. The data for each dog was collected on the same day. During the data collection, all dogs were allowed pain medications and supplements as per usual. Due to the FHO being obviously palpable, none of the observers were blinded for the FHO side.

### 2.1. Owner Questionnaire

Dog owners answered a written questionnaire in their native language (Finnish). In addition to signalment information (age, breed, and body weight), the questionnaire comprised 60 questions, 16 of which were used for the purpose of this study. These 16 questions were related to use of post-operative physiotherapy, current well-being, physical function, and the owner’s opinion of treatment outcome. Fourteen questions were in a multiple choice or binary format and two utilized the visual analogue scale (VAS).

The owners were asked about whether the dog had received physical therapy (i.e., range of motion or weight-bearing exercises, massage, swimming or hydrotherapy) (yes or no), how many times the dog received physiotherapy (1–5 times, 6–10 times, 11–15 times, 16–20 times, >20 times) and specifically whether the rehabilitation included hydrotherapy (swimming or underwater treadmill) (yes or no).

Six of the questions were related to the dog’s ability to walk, trot, gallop, jump, lie down, and get up from a lying position (very easily, easily, reasonably well, with difficulty, with great difficulty). The owners were asked whether the dog was weight bearing equally between the FHO and non-FHO limb (always, almost always, often, occasionally, rarely), whether the dog could sit normally (yes or no), and whether the dog was bunny hopping when galloping (very rarely, rarely, sometimes, often, very often). Two of the questions utilized a VAS. On the first VAS, the owners were asked to evaluate the dog’s movement by drawing an X on the VAS line at the point they think best describes the current situation. The length of the horizontal scale was 10 cm, where the ends of the line were defined as extreme limits; one end represented no difficulties whatsoever and the other end the worst possible situation. On the second VAS, the owners were asked to assess their dog’s quality of life by drawing an X on the line at the point they think best describes the current situation. One end of the line describes the best possible situation and the other end the worst possible situation. The VAS evaluation was quantified by measuring the place of the marking on the line with a ruler. One centimetre represented one grade on the scale. The placement of the X was measured with a ruler and graded accordingly, and the grades were used in the data analysis.

The owners were also asked whether the dog returned to original use/normal activities after the surgery (yes or no) and their evaluation of the outcome of treatment (excellent, good, fair, poor).

### 2.2. Orthopaedic Evaluation

The clinical orthopaedic examination was performed by an experienced veterinary orthopaedic surgeon (M.M.). The purpose of the examination was to exclude any other pathologies that might affect the variables examined in this study. The examination included a visual lameness assessment in walk and trot on a scale from 0 to 4 (no lameness, mild lameness or gait abnormality, moderate lameness or gait abnormality, severe weight-bearing lameness, non-weight-bearing lameness) [20] and a visual static weight-bearing assessment graded as normal or off-loading for each limb. All joints of the extremities were palpated and assessed for possible pain, swelling, instability, reduced range of motion, or crepitation, and all parameters were graded as normal, mild, moderate, or severe. The stifle joint was also assessed for possible medial or lateral patella luxation, graded from normal to grade 1 to 4 [21]. Possible craniocaudal instability by tibial compression test was graded as normal/negative or positive. The flexor withdrawal reflex was tested and graded as normal or decreased, and the lumbosacral area was palpated and graded as normal or painful.

### 2.3. Anatomical Measurements of Pelvic Position

Anatomical measurements of the pelvic position were performed by an experienced chartered physiotherapist specialized in veterinary physiotherapy (H.H.).

#### 2.3.1. Thigh Circumference

The thigh circumference was measured with a standard metric scale tape measurer and a spring tension device, with the dog placed in lateral recumbency. The measured limb was in a neutral, relaxed position. The measurements were taken mid-femur, measured between the greater trochanter and the lateral femoral condyle, i.e., at 50% of the length of the femur. The non-FHO thigh served as a control.

#### 2.3.2. Passive Range of Motion of the Coxofemoral Joint

Passive coxofemoral flexion and extension were measured with a standard transparent plastic goniometer with one-degree increments. The dog was positioned in lateral recumbency for the measurements. The axis of the goniometer was placed over the greater trochanter, the static arm was placed parallel to a line between the tuber sacrale and the tuber ischii, while the mobile arm was placed along the femoral longitudinal axis, between the greater trochanter and the lateral femoral epicondyle, as described previously [22,23]. Full available range was measured. The end of range was defined as a limitation in movement either by pain, discomfort, or mechanical tissue-related restrictions. The non-FHO limb served as a control.

#### 2.3.3. Static Weight Bearing

The distribution of static weight bearing between the limbs was measured with two identical, factory-calibrated, digital bathroom scales (Medica plus M-135, Truebell, Vantaa, Finland), which were used individually under each hind limb. The forelimbs were placed on a custom-made, non-slippery platform of the same height as the scales. The owner was instructed to hold the dog from the front, keeping it in a straight square standing position and not provide any support to the dog. The mean of the three measurements was used in statistical analysis. The protocol has been described previously [24].

#### 2.3.4. Measurements of Pelvic Position

**Figure 1:**
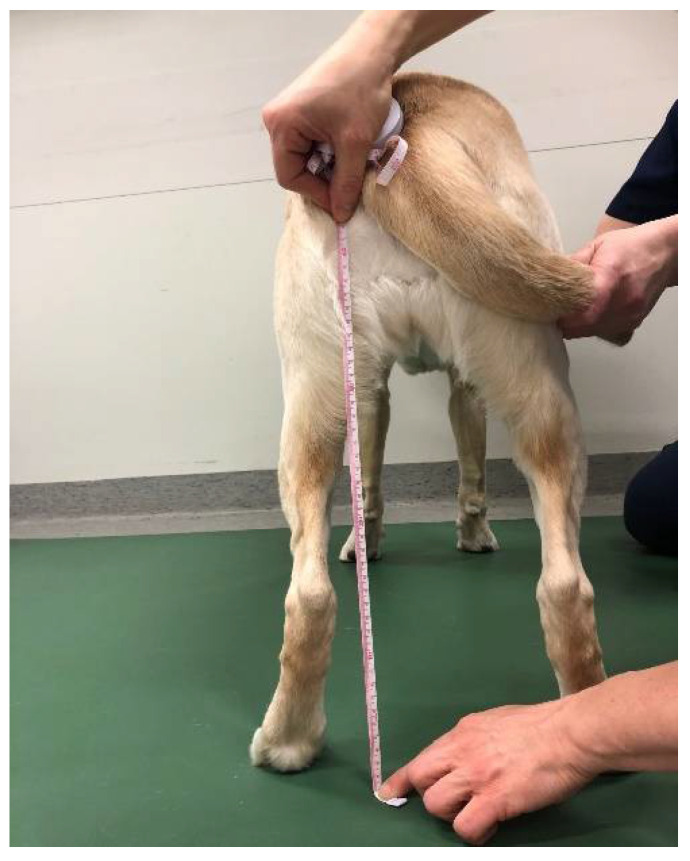
Measurement of the height of the tuber ischii from the ground with a standard tape measurer.

**Figure 2:**
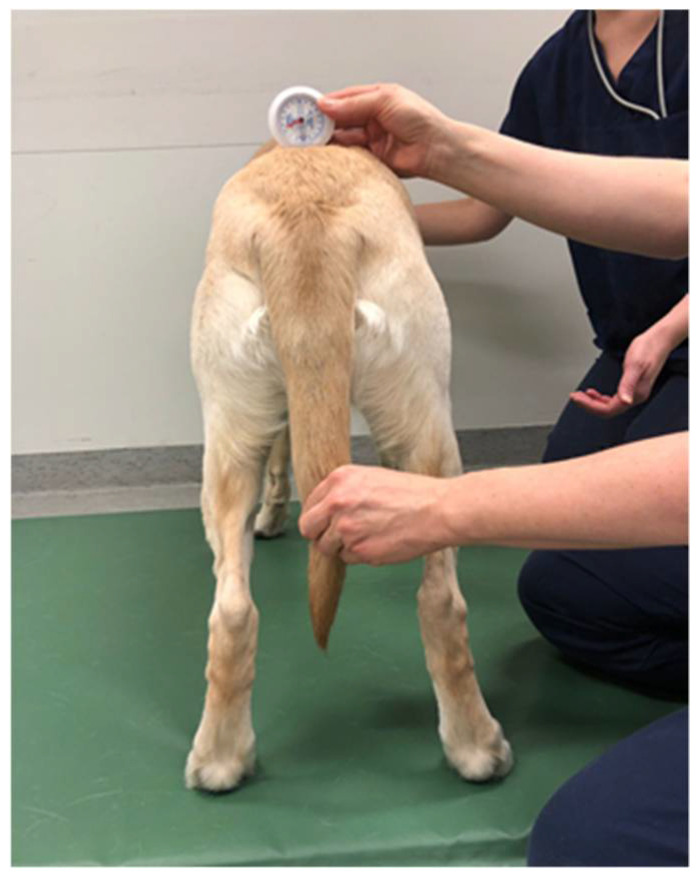
Measurement of the dorsoventral tilt of the pelvis with Myrin goniometer. Not visible in the image, attached behind and running under the Myrin, is a short plastic level reaching over the iliac crests, providing flat base for the Myrin.

The pelvic position was measured with the dog standing square and the owner standing in front, holding the head of the dog straight. The distance from the ground to the tuber ischii was determined using a tape measure with a metric scale (Figure 1). The dorsoventral tilt of the pelvis was measured at the point of tuber sacrale by using a Myrin gravitational goniometer (Follo Futura AS, Ås, Norway) (Figure 2). The Myrin gravitational goniometer consists of a small fluid-filled box fixed to a plate that is affected by the earth’s magnetic field and an inclination needle that is affected by gravity. Movement in the horizontal plane around a vertical axis was read from the compass needle. The degree of tilt was reported with a specificity of one degree.

### 2.4. Temporospatial Gait Parameters on Pressure-Sensitive Walkway

A pressure-sensitive walkway (GAITRight Electronic Walkway, Peekskill, NY, USA) was used to determine whether the dogs had temporospatial asymmetries in stance time, swing time, peak pressure, or hind limb reach. The walkway has an active area of 60.96 cm × 609.6 cm (90 cm × 700 cm total area), and an inactive 90 cm × 125 cm mat was placed at each end of the walkway to minimize any effect on movement caused by surface change. Accompanying software recorded and interpreted the pressure changes in the walkway sensors (GaitRite Manual V.3.9, CIR Systems, Sparta, NJ, USA). A scan rate of 240 Hz was used.

The dogs were first acclimatized to the walkway during one to three passes over it without any recordings. All dogs, led by their owners or an assistant, trotted four to six times over the walkway at a comfortable trotting speed, with no eye contact with the handler, no pull on the leash, and as freely as possible. Runs in both directions were recorded, and data of at least eight full gait cycles from at least three separate valid runs were collected. Mean values were used in statistical analysis according to a previous protocol [20].

### 2.5. Statistical Methods

Descriptive analysis was performed in IBM SPSS Statistics for Windows, version 25.0 (IBM Corp., Armonk, NY, USA) and Excel for Microsoft 365 (Microsoft Corp., Redmond, WA, USA). All other analyses were conducted with SAS System for Windows, version 9.4 (SAS Institute Inc., Cary, NC, USA). 

The differences between the FHO and non-FHO limbs in height of the tuber ischii, static weight bearing, thigh circumference, coxofemoral flexion, coxofemoral extension, stance time, swing time, peak pressure, and hind limb reach were analysed utilizing linear mixed effects models. Each variable was analysed separately. The models included the variable in question as the response, operation status (yes vs. no) as the sole fixed effect, and dog as the random effect. The differences between the FHO and non-FHO limb were estimated from the models with least square means and their 95% confidence intervals. Static weight bearing was evaluated as percentages proportional to the total weight of the dog to consider the different sizes of the ten dogs as previously reported [24]. The static weight-bearing measurements also included one clear outlying dog (dog number ten). The outlying dog was excluded from the final analysis of static weight bearing.

The differences between the front and hind limbs within the FHO and non-FHO side of each dog in stance time and swing time were analysed similarly as the comparisons between the hind limbs but using the limb location (front vs. hind) as the sole fixed effect instead of the operation status.

Model fits were assessed by evaluating studentized model residuals graphically (normal QQ-plots) and by tests of normality (Shapiro–Wilk). *p*-values of <0.05 were considered significant in all analyses.

All mean values are reported together with standard deviation.

## 3. Results

Ten dogs, three males and seven females, met the inclusion criteria. Seven of the dogs had had left and three right FHO. The dogs´ median weight was 8.0 kg, with the lightest weighing 4.2 kg and the heaviest 24.8 kg. The median age when the surgery was performed was 1.4 years; the youngest patient was 0.6 years and the oldest 6.4 years. The study was performed at a median of 2.5 years since the FHO, with the earliest at 0.9 years and the latest at 4.8 years. The following breeds were represented: Border Terrier (*n* = 1), Japanese Chin (*n* = 1), Japanese Spitz (*n* = 1), German Shepherd (*n* = 1), Parson Russell Terrier (*n* = 1), Wirehaired Fox Terrier (*n* = 1), and mixed breed dogs (*n* = 4). All dogs were family dogs/pets. Two of the dogs were on pain medication, and two of the dogs were on joint supplements at the time of data collection. 

### 3.1. Questionnaire

All dogs received post-operative physiotherapy as recommended in the hospital protocol post-FHO. Five dogs had less than five rehabilitation sessions, two dogs had six to ten sessions, and three dogs had 16–20 sessions. Seven of the dogs received hydrotherapy in addition to other forms of physiotherapy, such as range of motion or weightbearing exercises and massage or manual mobilization.

The owners reported that at the time of the study eight of ten dogs walked and trotted very easily or easily. Nine of ten dogs galloped and jumped very easily or easily. Nine dogs lay down very easily or easily, and ten dogs got up from lying very easily or easily. Seven dogs were weight bearing equally always or almost always. Nine dogs were considered to sit normally. Bunny hopping was observed in four dogs very rarely or sometimes while galloping, while six dogs bunny hopped often or very often.

Nine dogs returned to original use/normal activity level after the FHO. The owners estimated their dogs´ movements as mean 1.3 (±0.9) on a ten-graded VAS, where zero represented no difficulties whatsoever and ten the worst possible situation. The quality of life was estimated as mean 1.0 (±0.6) on the same scale, where zero represented the best possible and ten the worst possible. Six owners considered the outcome of treatment to be excellent and four good.

### 3.2. Orthopaedic Evaluation

The evaluation was performed to rule out any other pathologies that might affect the study variables. Three of the dogs had grade 1 lameness in the FHO limb in walk and trot, while one dog had grade 1 lameness in trot only. None of the dogs seemed to offload any limb in the visual static weight-bearing assessment. Four of the dogs had reduced extension in the hip joint of the FHO limb. One dog had bilateral grade 2 patella luxation. One dog had grade 2 patella luxation on the FHO limb and grade 3 on the non-FHO limb. One dog had grade 2 patella luxation on the FHO limb, and one dog grade 2 patella luxation on the non-FHO limb. There were no other relevant findings. All dogs were deemed able to participate in the study.

### 3.3. Anatomical Measurements

#### 3.3.1. Thigh Circumference

The thigh circumference was significantly (estimate 1.60 cm) less in the FHO limb (*p* = 0.005) with least square mean (LSM) 20.2 (95% confidence interval 16.4; 23.9) cm in the FHO limb and 21.8 (18.0; 25.5) cm in the non-FHO limb. There was no significant difference (*p* = 0.39) in the height of the tuber ischii from the ground (Table 1). The measurement of tuber sacrale levels with the Myrin gravitational goniometer showed a dorsoventral tilt of 1–2 degrees, with five dogs tilting towards the FHO side and five dogs tilting away from the FHO side.

The two dogs with bilateral patella luxation had less thigh circumference in the FHO limb: 16 cm and 13 cm in the FHO limb, versus 20 cm and 14 cm in the non-FHO limb, respectively. The dog with patella luxation in the non-FHO limb had less thigh circumference in the FHO limb: 17 cm in the FHO limb versus 18.5 cm in the non-FHO limb. The dog with patella luxation in the FHO limb had equal thigh circumference in both hind limbs: 19 cm. All of the dogs with patella luxation had equal or smaller thigh circumference in the FHO limb, when compared to the non-FHO limb, despite their patellar luxation status.

#### 3.3.2. Static Weight Bearing

There was a significant difference (*p* = 0.031) in the static weight bearing between the FHO and non-FHO limbs, where the LSM value of the FHO limb was 15.6% (12.7; 18.5) of the total body weight versus 20.3% (17.4; 23.2) of the total body weight of the non-FHO limb, as presented in Table 1.

#### 3.3.3. Range of Motion

There was no significant difference in coxofemoral flexion on the FHO limb (LSM 45.5 (41.4; 49.6)) compared to the non-FHO limb (LSM 43.0 (38.9; 47.1)) (*p* = 0.21). Regarding extension, there was a significant difference (*p* = 0.003): 21.5 degrees less on the FHO limb (LSM 128.5 (117.4; 139.6) degrees) than on the non-FHO limb (LSM 150.0 (138.9; 161.1) degrees) (Table 1).

### 3.4. Temporospatial Gait Parameters on Pressure-Sensitive Walkway

Significant differences emerged between front and hind limbs in swing time and stance time in trot (Table 2) for both FHO and non-FHO sides. There was, however, no significant difference between the FHO and non-FHO limb either in stance time (*p* = 0.70), swing time (*p* = 0.26) or peak pressure (*p* = 0.91) in trot. There was numerically more hind limb reach in the FHO limb, −1.0 (−2.54; 0.44) cm, than in the non-FHO limb, −1.6 (−3.12; −0.15) cm, albeit non-significant (*p* = 0.14) (Table 1).

When descriptively comparing the stance time on the FHO and non-FHO sides, values were almost identical on both front limbs and on both hind limbs. The same trend was seen in swing time.

## 4. Discussion

Our study is the first one, to our knowledge, to report information regarding static weight bearing of dogs who have had FHO. We also provide new information regarding temporospatial values in trot of these dogs. This study also presents a new method of quantifying pelvic position in dogs: the Myrin goniometer. In addition to this new information, the study concurs previously published findings regarding limited hip range of motion and hind limb muscle mass asymmetry in these dogs.

Eight of ten dogs in our study had a smaller thigh circumference in the FHO limb than in the non-FHO limb. This result is in line with previously published studies, where 50% of 127 dogs and 82% of 66 dogs also had muscle atrophy after FHO [13,15], respectively. This is clinically noteworthy, since the dogs in our study were measured a mean of 2.7 years after surgery. The muscle atrophy thus seems to be a long-term consequence. The reduced thigh circumference might be a consequence of chronically decreased range of motion and weight bearing of the limb. 

Static weight bearing was, on average, 15.6% of the total body weight on the FHO limb versus 20.3% on the contralateral limb, which sums close to the 37.5% reported to be a normal front/hind body weight distribution ratio in dogs [25], with breed variations from 31% to 38% [26]. This indicates that the dogs are mainly offloading weight to the contralateral hind limb, and not so much to the front limbs. Reasons for weight offloading from the FHO limb in standing could be pain, weakness or limb-length difference [7,10,13,15]. Another possible reason might be altered muscle activation patterns. To account for these reasons, the dogs in our study were assessed by a veterinary orthopaedic surgeon and were deemed free of pain, despite the asymmetrical weight bearing finding. Assessment of muscle activation was not within the scope of this study, but further research on this topic is warranted, as muscle function has been shown to play an important role in, for example, stifle function [27].

Coxofemoral flexion was not affected by the FHO, whereas a significant decrease occurred in FHO-affected coxofemoral extension. This follows the acknowledged pattern of extension being first and foremost affected after FHO, hip dysplasia, and hip osteoarthritis [18,28]. In our study, eight of ten dogs had reduced extension in the FHO limb at a mean of 2.7 years after the operation, which is comparable with another study where 74% of 66 dogs had restricted coxofemoral movement at a mean of four years [15]. Less than 10 degrees loss of joint motion will likely not impact the limb function, while more severe restrictions will probably affect the dog´s ability to trot, gallop, and jump [18]. For comparison, normal passive coxofemoral range of motion in healthy Labrador retrievers has been reported to be 50 degrees in flexion and 162 degrees in extension [22], while active flexion in trot on a treadmill is measured at 97 degrees and active extension 124 degrees [29], which is not near the maximal range. The dogs in this study had a mean of 21.5 degrees less extension in the FHO limb, which can explain the bunny hopping reported by the owners. However, bunny hopping when galloping could also be related to pain, discomfort, or weakness.

We expected the temporospatial values of trot to be affected by the FHO. Specifically, we anticipated seeing a shorter stance phase on the FHO limb, which has been noticed previously when dogs had a shortened ground contact time even though visual lameness was not always detected [15]. Remarkably, despite the above-described findings of less static weight bearing, muscle mass, and coxofemoral extension in the FHO limb, there were no significant findings in most locomotion-related factors (swing time, stance time, peak pressure), and the dogs trotted normally. When comparing stance time and swing time between front and hind limbs, the time distributions follow the same pattern as in healthy dogs [30]. This suggests that the dog manages to compensate the chronic FHO-related shortcomings during trot.

According to the questionnaire responses, owner satisfaction with FHO-related outcomes was high, which is supported by the findings of previous studies [13,14,15]. Most of the dogs regained function and performed their daily activities very easily or easily. All of the dogs in our study received physiotherapy, with a combination of home exercises and treatments by a physiotherapist, working with individually adjusted rehabilitation programmes. However, the content and detail regarding the physiotherapy that was undertaken in each of the dog’s case was not available to us. Commonly modern rehabilitation programmes generally aim at gaining coxofemoral extension, muscle mass, and active use of the operated limb [18]. Many of the earlier studies on outcomes of FHO are older [13,14,15], when post-operative physiotherapy was not yet used as it is today. Thus, it is important to consider the possible effect of therapy on the outcome compared with the outcomes reported previously. Nevertheless, we did not have a control group, and therefore, no conclusion of the effect of physiotherapy can be drawn, as we do not know whether dogs not receiving physiotherapy would have had more or less findings than the findings in our group. 

We used two different methods to measure the pelvic position in standing: height of the tuber ischii and dorsoventral tilt of the tuber sacrale. Measurements of the tuber ischii did not show any significant tilt of the pelvis, while the Myrin gravitational goniometer showed a tilt to the FHO side in five dogs and away from the FHO side in five dogs. We assumed that the results of the two methods would have agreed, but they did not. We assumed the dog would perform a dorsoventral tilt in the axial plane and there would be a correlating difference in tuber ischii and tuber sacrale. The dogs might have changed position between the two measurements, although we did try to control the measurement positions. In addition, the normal distribution of natural asymmetry in healthy dogs is not known. We also do not know how many degrees tilt in the tuber sacrale would be equivalent to a certain difference in height in the tuber ischii. The Myrin gravitational goniometer might be a more sensitive instrument for measuring pelvic tilt than the tuber ischii-based tape measurement method. In a previous study, a tilt away from the operated side was seen in 38% of cases 8 years post surgery [13]. However, the method of measurement and how much the pelvis tilted or if the pelvis was horizontal in 62% of dogs were not provided [13]. Shortening of the limb or displacement of the proximal femur has been used to explain pelvic tilt towards the FHO side [12,15], while a tilt away would not be able to compensate for any limb shortening [13]. However, a tilt away from the FHO side could correlate better with reduced static weight bearing. The Myrin gravitational goniometer is frequently used in humans and is considered valid and reliable when measuring human cervical movement [31,32]; however, to our knowledge, it has not previously been utilized in animals. It could provide a new measurement method in veterinary medicine, but validation studies are needed. The fact that the Myrin gravitational goniometer has not been reliability tested or examined for its validity when used on dogs, specifically for measuring their pelvic position, could, of course, have affected our results. However, it should be noted that the basic rules of using the tool for such measurements can be applied from humans, where the tool is routinely used.

The outcome of FHO is often considered to be associated with the size of the dog [33], with the perception that smaller dogs and cats cope more easily with the absence of the coxofemoral joint [34]. The dogs in our study had a median weight of 8.0 kg, which could contribute to the non-affected gait and temporospatial parameters in a positive way. A larger dog puts more weight on the pseudo-joint, which could cause more pain and malplacement of the proximal femur, negatively affecting the gait. One theory is also that a larger dog has more excessive bone remodelling, bone-on-bone contact between the femoral neck and acetabulum, and bone lysis, which is thought to cause excessive pain and lameness [33]. However, there are studies that indicate that the weight of dogs could be of lesser importance to the outcome than commonly assumed [35,36].

### Limitations of the Study

Although several papers have been published regarding the outcome of FHO, our study continues to contribute to the somewhat contradictory existing information. In addition, we combine several outcome measurement results—some never previously published—offering information of the functionality of these patients. However, this study was not without limitations.

In our study, sample selection bias is possible. The study utilized a convenience sample; all available dogs meeting the inclusion criteria were included. However, the sample size was not as large as originally desired. Due to the small number of dogs participating in the study, there is a risk for the positive outcome of surgery to be over-represented. 

Having a larger sample size would have allowed us to be more selective with the dogs that were included in the study. Using the contralateral limb as a control is a weakness of the study, especially when it was not completely healthy. Bilateral FHO was excluded for this reason. Four of the included dogs had unilateral or bilateral medial patella luxation that could have possibly affected the result. Two dogs had bilateral patella luxation, one had patella luxation in the FHO limb, and one had patella luxation in the non-FHO limb. In theory, the possible effect of these could be considered to cancel each other out. Moreover, the inclusion of the individuals with the patella luxation was based on the assessment that the condition was not painful or affected the gait, as concluded in the orthopaedical assessment of the patients. This was, in the end, also confirmed by measuring the thigh circumference, which was either equal or smaller in the FHO limb. Similar findings were seen in the temporospatial gait parameters. 

Ideally, individuals with no other orthopaedic disease would have been used. However, in reality, it is very rare to have a patient requiring FHO and having no other orthopaedical issues. As this study was a clinical study, aiming to assess the rehabilitation of FHO patients in a non-controlled environment, it was felt that the selection of animals was a representation of the clinical reality of the target group that the clinicians deal with on daily basis.

Moreover, large variation existed in age, breed and size of the dogs included. However, most dogs in the study were small or medium-sized. Larger dogs could have had a less positive outcome from the surgery. The contralateral limb was used as a control, as no control group with individuals not receiving postoperative physiotherapy was available. Moreover, recall bias is possible when a median time of 2.5 years had passed between the FHO and owners completion of the questionnaire. The owners perception of their dogs rehabilitation and recuperation may have been affected by time, thus not being completely accurate. However, this is a weakness in all owner questionnaires, and the information gained is still considered to be valuable.

Regarding the timing of the data collection, it should be stated, that the data was collected at two different occasions, with 5 months in between, which can also have affected the results of the assessments made by the authors. 

Another limitation is that although an orthopaedic examination was performed, the spinal structures were not examined by imaging. There may have been pathologies in the vertebral column, which went unnoticed in the clinical examination, but may still have affected the position of the pelvis. This may be one of the reasons for the lack of a pattern in pelvic position in these dogs. Moreover, a complete neurological evaluation was not performed. 

## 5. Conclusions

After more than six months from FHO, the affected limb has muscle atrophy, decreased coxofemoral extension, and static weight bearing, despite dog owners considering the result of the FHO to be excellent or good. However, hind limb stance and swing time, peak pressure and hind limb reach in trot does not seem to be affected by the anatomical limitations. In our group of dogs, there was no specific pattern of pelvic position due to the FHO.

## Figures and Tables

**Table 1:** Differences between FHO and non-FHO limbs analysed with linear mixed effects models presented as least square means (LSMs) and their 95% confidence intervals (CIs).

Measurements	N	LSM (95% CI) FHO	LSM (95% CI) Non-FHO	Difference (95% CI)	*p*-Value
Height of tuber ischii (cm)	10	25.3 (19.2; 31.4)	25.1 (19.0; 31.2)	0.22 (−0.33; 0.77)	0.39
Static weightbearing (%)	9	15.6 (12.7; 18.5)	20.3 (17.4; 23.2)	−4.69 (−8.82; −0.56)	0.031 *
Thigh circumference (cm)	10	20.2 (16.4; 23.9)	21.8 (18.0; 25.5)	−1.60 (−2.58; −0.62)	0.005 *
Hip flexion (degrees)	10	45.5 (41.4; 49.6)	43.0 (38.9; 47.1)	2.50 (−1.71; 6.72)	0.21
Hip extension (degrees)	10	128.5 (117.4; 139.6)	150.0 (138.9; 161.1)	−21.5 (−33.5; −9.5)	0.003 *
Stance time (s)	10	0.12 (0.09; 0.15)	0.12 (0.09; 0.15)	0.004 (−0.017; 0.025)	0.70
Swing time (s)	10	0.27 (0.24; 0.31)	0.26 (0.23; 0.30)	0.009 (−0.008; 0.026)	0.26
Peak pressure (N)	10	14.7 (8.5; 20.9)	14.6 (8.4; 20.8)	0.068 (−1.30; 1.44)	0.91
Hind reach (cm)	10	−1.05 (−2.54; 0.44)	−1.64 (−3.12; −0.15)	0.58 (−0.22; 1.39)	0.14

Significance indicated with an asterisk (*), N = number of dogs.

**Table 2:** Differences between front and hind limbs on FHO and non-FHO sides analysed with linear effect models presented as least square means (LSMs) and their 95% confidence intervals (CIs).

Measurement	N	LSM (95% CI) Front	LSM (95% CI) Hind	Difference (95% CI)	*p*-Value
Stance time FHO side (s)	10	0.15 (0.12; 0.17)	0.12 (0.09; 0.15)	0.025 (0.010; 0. 039)	0.0038 *
Stance time non-FHO side (s)	10	0.14 (0.12; 0.17)	0.12 (0.09; 0.14)	0.026 (0.017; 0.036)	0.0002 *
Swing time FHO side (s)	10	0.27 (0.20; 0.27)	0.27 (0.24; 0.31)	−0.035 (−0.039; −0.031)	0.0001 *
Swing time non-FHO side (s)	10	0.23 (0.20; 0.27)	0.26 (0.23; 0.29)	−0.028 (−0.040; −0.016)	0.0005 *

Significance indicated with an asterisk (*), N = number of dogs.

## Data Availability

Data are not available due to ethical and privacy limitations based on the consent provided by participants.

## References

[1] Piermattei D.L., Flo G.L., DeCamp C.E. (2006). Chapter 16—The hip joint. Handbook of Small Animal Orthopaedics and Fracture Repair.

[2] Schiller T.D. (2017). BioMedtrix total hip replacement systems: An overview. Vet. Clin. Small Anim..

[3] Hummel D. (2017). Zurich cementless total hip replacement. Vet. Clin. Small Anim..

[4] Anderson A. (2011). Treatment of hip dysplasia. J. Small Anim. Pract..

[5] Petazzoni M., Tamburro R. (2022). Clinical outcomes of double pelvic osteotomies in eight dogs with hip dysplasia aged 10–28 months. Vet. Surg..

[6] Prostredny J.M., Bojrab A.J., Waldron D.R., Toomb J.P. (2014). Excision arthroplasty of the femoral head and neck. Current Techniques in Small Animal Surgery.

[7] Harper T.A.M. (2017). Femoral head and neck excision. Vet. Clin. Small Anim..

[8] Rawson E.A., Aronsohn M.G., Burk R.L. (2005). Simultaneous bilateral femoral head and neck ostectomy for the treatment of canine hip dysplasia. J. Am. Anim. Hosp. Assoc..

[9] Sabiza S., Ronagh A., Khajeh A. (2019). Effective medical management and physiotherapy program of femoral head and neck ostectomy in 24 dogs and cats; clinical report. Iran. J. Vet. Surg..

[10] Davidson J.R., Kerwin S., Millis D.L., Levine D. (2014). Chapter 32—Common Orthopaedic conditions and their physical rehabilitation. Canine Rehabilitation and Physical Therapy.

[11] Wittek K., Bockstahler B., Vannini R., Reicher B., Mucha M., Maierl J., Bockstahler B. (2019). Chapter 28—Treatment plans. Essential Facts of Physical Medicine, Rehabilitation and Sports Medicine in Companion Animals.

[12] Harasen G. (2004). The femoral head and neck ostectomy. Can. Vet. J..

[13] Duff R., Campbell J.R. (1977). Long term results of excision arthroplasty of the canine hip. Vet. Rec..

[14] Berzon J.L., Howard E., Covell S.J., Trotter E.J., Dueland R. (1980). A retrospective study of the efficacy of femoral head and neck excisions in 94 dogs and cats. Vet. Surg..

[15] Off W., Matis U. (2010). Excision arthroplasty of the hip joint in dogs and cats. Vet. Comp. Orthop. Traumatol..

[16] Planté J., Dupuis J., Beauregard G., Bonneau H.H., Breton L. (1997). Long-term results of conservative treatment, excision arthroplasty and triple pelvic osteotomy for the treatment of hip dysplasia in the immature dog Part 2: Analysis of the ground reaction forces. Vet. Comp. Orthop. Traumatol..

[17] Grisneaux E., Dupuis J., Pibarot P., Bonneau N.H., Charette B., Blais D. (2003). Effects of postoperative administration of ketoprofen or carprofen on short- and long-term results of femoral head and neck excision in dogs. J. Am. Vet. Med. Assoc..

[18] Dycus D., Levine D., Marcellin-Little D.J. (2017). Physical rehabilitation for the management of canine hip dysplasia. Vet. Clin. Small Anim..

[19] Miyata T., Kawamura K. (2019). Rehabilitation treatment for long-term associated femoral head ostectomy muscle atrophy. Vet. Rec. Case Rep..

[20] Mölsä S.H., Hyytiäinen H.K., Morelius K.J., Palmu M.K., Pesonen T.S., Lappalainen A.K. (2020). Radiographic findings have an association with weight bearing and locomotion in English bulldogs. Acta Vet. Scand..

[21] Farell M. (2018). Chapter 23—The Stifle. BSAVA Manual of Canine and Feline Musculoskeletal Disorders: A Practical Guide to Lameness and Joint Disease.

[22] Jaegger G., Marcellin-Little D.J., Levine D. (2002). Reliability of goniometry in Labrador Retrievers. Am. J. Vet. Res..

[23] Nicholson H.L., Osmotherly P.G., Smith B.A., McGowan C.M. (2007). Determinants of passive hip range of motion in adult greyhounds. Aust. Vet. J..

[24] Hyytiäinen H., Mölsä S.H., Junnila J., Vapaavuori O., Hielm-Björkman A.K. (2012). Use of bathroom scales in measuring asymmetry of hind limb static weight bearing in dogs with osteoarthritis. Vet. Comp. Orthop. Traumatol..

[25] Phelps H.A., Ramos V., Shires P.K., Were S.R. (2007). The effect of measurement method on static weight distribution to all legs in dogs using the Quadruped Biofeedback System. Vet. Comp. Orthop. Traumatol..

[26] Humphries A., Shaheen A.F., Gómez Álvarez C.B. (2020). Biomechanical comparison of standing posture and during trot between German shepherd and Labrador retriever dogs. PLoS ONE.

[27] Adrian C.P., Haussler K.K., Kawcak C., Reiser R.F., Riegger-Krugh C., Palmer R.H., McIlwraith C.W., Taylor R.A. (2013). The role of muscle activation in cruciate disease. Vet. Surg..

[28] Farrell M., Clements D.N., Mellor D., Gemmill T., Clarke S.P., Arnott J.L., Bennett D., Carmichael S. (2007). Retrospective evaluation of the long-term outcome of non-surgical management of 74 dogs with clinical hip dysplasia. Vet. Rec..

[29] Agostinho F.S., Rahal S.C., Miqueleto N.S.M.L., Verdugo M.R., Inamassu L.R., El-Warrak A.O. (2011). Kinematic analysis of Labrador retrievers and Rottweilers trotting on a treadmill. Vet. Comp. Orthop. Traumatol..

[30] Kano W.T., Rahal S.C., Agostinho F.S., Mesquita L.R., Santos R.R., Monteiro F.O., Castilho M.S., Melchert A. (2016). Kinetic and temporospatial gait parameters in a heterogeneous group of dogs. Vet. Rec..

[31] Hagen K.B., Harms-Ringdahl K., Enger N.O., Hedenstad R., Morten H. (1997). Relationship between subjective neck disorders and cervical spine mobility and motion-related pain in male machine operators. Spine.

[32] Won Y.K.Y., Latip H.F.M., Aziz M.S.A. (2019). The reliability and validity on measuring tool of cervical range of motion: A review. Sport Med. Inj. Care.

[33] Ober C., Pestean C., Bel L., Taulescu M., Milgram J., Todor A., Ungur R., Leșu M., Oana L. (2018). Use of clinical and computed tomography findings to assess long-term unsatisfactory outcome after femoral head and neck osteotomy in four large breed dogs. Acta Vet. Scand..

[34] Fitzpatrick N., Pratola L., Yeadon R., Nikolaou C., Hamilton M., Farrell M. (2012). Total hip replacement after failed femoral head and neck excision in two dogs and two cats. Vet. Surg..

[35] Montgomery R.D., Milton J.L., Horne R.D., Coble R.H., Williams J.C. (1987). A retrospective comparison of three techniques for femoral head and neck excision in dogs. Vet. Surg..

[36] Fattahian H., Mohyeddin H., Hoseinzadeh A., Akbarein H., Moridpour R. (2012). Excision arthroplasty of the hip joint in dogs: The role of age, weight, degenerative joint disease on the outcome. Kafkas Univ. Vet. Fak. Derg..

